# Effects of Adding Ketamine to Fentanyl Plus Acetaminophen on Postoperative Pain by Patient Controlled Analgesia in Abdominal Surgery

**DOI:** 10.5812/aapm.12162

**Published:** 2013-12-26

**Authors:** Farnad Imani, Hamid Reza Faiz, Minow Sedaghat, Maryam Hajiashrafi

**Affiliations:** 1 Department of Anesthesiology, Rasoul Akram Medical Center, Iran University of Medical sciences, Tehran, Iran

**Keywords:** Ketamine, Acetaminophen, Fentanyl, Postoperative, Pain, Abdomen, Surgical Abdomen

## Abstract

**Background:**

Postoperative pain is one of the most important complications encountered after surgery. A number of options are available for treating pain following surgery. One of those options is the use of intravenous patient-controlled analgesia (PCA). Ketamine is an anesthetic drug relieving pain with its NMDA receptor antagonistic effect.

**Objectives:**

This study is aiming at better pain management after abdominal surgery; the effects of adding ketamine to intravenous fentanyl plus acetaminophen PCA were evaluated.

**Patients and Methods:**

In a double-blind randomized clinical trial 100 patients, ASA I or II, 20 - 60 years old were divided into two groups. These patients were abdominal surgery candidates. In order to control postoperative pain in the control group an IV patient-control analgesia (PCA) containing fentanyl 10 μg/mL plus acetaminophen 10 mg/mL was instructed to be used for the patients, but the patients in ketamine group received ketamine 0.5 mg/mL plus control group PCA content. During the first 48 hours after surgery, ketamine patients were evaluated every 8 hours (at rest, while moving and coughing) to determine their pain scores using VAS scale, sedation score, additional analgesics, nausea and vomiting.

**Results:**

There were no significant demographic differences between two groups. Pain scores (at rest, while moving and coughing) during the first 48 hours were not significantly different between two groups (P values = 0.361, 0.367 and 0.204, respectively). Nausea scores were significantly lower in the ketamine group (P = 0.026).

**Conclusions:**

The addition of ketamine to intravenous fentanyl plus acetaminophen PCA had not extra effects in relieving post abdominal surgery pain.

## 1. Background

Postoperative pain is considered as one of the most important problems encountered by the patients after abdominal surgery. 

A Physician may employ various treatments to control the pain (1). That is why various methods of analgesia are particularly significant. Oral or parenteral analgesics administration, preemptive analgesia (Gabapentin and Pregabalin), bupivacaine infiltration, continuous epidural anesthesia with catheters, peripheral nerve block and patient-controlled analgesia (PCA) are various treatment methods (2-8).

Poor postoperative analgesia occurred in different reasons such as extensive range of interpatient/intrapatient requirements in analgesia, serum drug levels (particularly in intramuscular injections) and delays in administration (9). Usual PRN analgesic procedures can hardly make up for these influences. Intravenous patient-controlled analgesia can avoid some complications in the procedure, enhance opioid delivery and diminish the influence of pharmacokinetic/pharmacodynamic factors in patients (9). Intravenous PCA works on the belief that there is a negative response cycle; the patients who self-administered the drug when they felt pain and when their pain relieved, no need drug is required (9). Ignoring this negative response cycle may be resulted in disproportionate sedation or respiratory depression. There have been some reports regarding equipment malfunctions but the PCA device itself usually works fine and mostly users or operators are responsible for the mistakes in using PCA (9).

Some analgesic techniques use certain medicines to improve the effectiveness of opioids. For instance, they benefit from adding chlorpromazine, promethazine, midazolam and clonidine to morphine PCA (10).

By triggering N-methyl-D-aspartate (NMDA) receptor, nociceptive stimulation can generate spinal cord hyperexcitability. Spinal cord hyperexcitability plays a considerable role in severe pain pathophysiology. Administering excessive doses of opioids can trigger the NMDA pain facilitating procedures leading to hyperalgesia. It also might relieve the pain after surgery. Consequently, the NMDA antagonist ketamine can play a part in the management of pain after surgery (11).

Ketamine is an anesthetic drug (9) with N-Methyl-D-aspartic acid (NMDA) receptor , which its antagonistic effect inhibits or reverses Central Nervous System (CNS) sensitivity to painful stimuli leading to pain relief after surgery (12-15).

## 2. Objectives

The aim of the present study is to investigate the effects of adding ketamine to intravenous fentanyl plus acetaminophen in patient controlled analgesia (PCA) on controlling pain after abdominal surgeries.

## 3. Patients and Methods 

After ethical approval, 100 patients were divided randomly into two groups to evaluate the postoperative pain control in a double-blinded clinical trial.

ASA physical status 1 and 2, 20 - 60 years old and candidates for abdominal surgery under general anesthesia.

Exclusion criteria: current drug abuse, allergy to medications, alcohol and psychotropic drugs abuse, pregnancy and lactation or not using a reliable method of contraception by women, patients who refused and failed to cooperate in the study, impaired liver function or liver enzyme disorders, a history of seizures, psychological disorders, chronic pain or daily use of analgesics, contraindications of the drug under study, and concurrent use of drugs or enzyme inducers.

General anesthesia methods were the same in all subjects. After IV administration of fentanyl (3 μg/kg) and midazolam (1 mg) for premedication, thiopental (5 mg/kg), cisatracurium (0.2 mg/kg) and remifentanil (1 μg/kg) were used to induce anesthesia. In order to maintain anesthesia, propofol (100 mg/kg/min) and remifentanil (1 - 2 mcg/kg/min) and repeated doses of 2 mg cisatracurium (every 30 minutes) were administered.

After the surgery and return of breathe to the normal level, reversal of atracurium with neostigmine (0.04 mg/kg) and atropine (0.02 mg/kg) was done and the patients were extubated after regaining consciousness and were taken to the recovery room where they were all hemodynamically monitored until fully achieving recovery room discharge criteria.

For the control group, each mL of the PCA contained fentanyl (10 μg) and acetaminophen (10 mg) but the patients in the ketamine group received ketamine 0.5 mg/mL plus control group PCA content. Infusion rate was set to 4 mL/h.

To have a “double-blind” study, intravenous PCAs (similar in appearance, labeled with A and B) administered the contents of which were only known to the project observer. In order to relieve post abdominal surgery pain, the patients were instructed the use of PCA in the recovery room. IV PCA administered before the onset of pain, and maximum 2 hours after the surgery. We informed patients about visual analogue scale (VAS from zero (no pain) to 10 (unbearable pain)). The patients were evaluated every 8 hours during the first 48 hours (at rest, while moving and coughing). Other parameters assessed included sedation score (Ramsay score from 1 to 6 shown in Table 1), psychological-nervous system complications and nausea (grading from 0 to 4) and/or vomiting (grading from 0 to 4). (Table 1) The category pruritus scores ranged from zero (no itching) to, 1 (mild itching) and 2 (severe itching). 

**Table 1.** Ramsay Sedation Scale and Nausea /Vomiting Grads

	Grade
**Ramsay scale**	
Patient anxious and agitated or restless, or both	1
Patient co-operative, oriented, and tranquil	2
Patient responds to commands only	3
Brisk response to a light glabellar tap or auditory stimulus	4
Sluggish response to a light glabellar tap or auditory stimulus	5
No response to the stimuli mentioned in items 4 and 5	6
**Nausea scale**	
Non –symptomatic	0
Loss of appetite without alteration in eating habits	1
Oral intake decreased without significant weight loss	2
Inadequate oral caloric or fluid intake	3
Life threatening consequences	4
**Vomiting scale**	
No emesis	0
1 episode in 24 h	1
2-5 episodes in 24 h	2
≥6 episodes in 24 h	3
Life threatening consequences	4

When pain scores exceeded from 3, acetaminophen 500 mg was administered intravenously and nausea and vomiting were controlled by administration of 4 mg ondansetron. If the patients were sedated (Ramsay sedation score 1) and showed neurological and psychological complications, the medication was discontinued.

The data obtained were analyzed using the statistical software SPSS 15. Quantitative data are shown as mean and qualitative data was shown as frequency. In case of normal distribution in both groups, Chi-square test and independent t-test were used to analyze qualitative variables and quantitative data respectively. Furthermore, due to repeated variables measurements and minimize the alpha error, the repeated measure test was applied. In this study P value was 0.05.

## 4. Results

**Figure 1.**
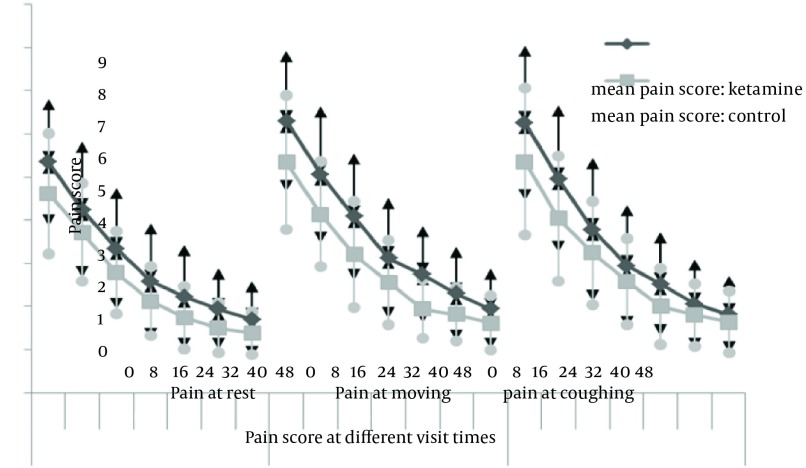
Pain Scores at T_0 _, T_0 _+ 8, 16, 24, 32, 40 and 48

One hundred patients were entered into the study. The difference in demographic data was not significant between the two groups (P values are 0.137, 0.159, and 0.153, respectively) (Table 2). T_0 _was the time of the patient arrival into the recovery room. Two patients in the ketamine group received 500 mg IV Acetaminophen at the 8th hour which was not statistically significant. Changes in pain scores (at rest, during movement and while coughing) were not significantly different between the two groups (P values were 0.361, 0.367 and 0.204, respectively) (Figure 1). 

**Table 2.** Demographic Features

	Ketamine	Control
**Female to male ratio**	46/4	41/9
**Age, Mean (SD), y**	43.98 (11.33)	48.82 (9.447)
**Surgery frequency**		
Colectomy	3	0
Cholecystectomy	14	15
Epigastric hernia	2	0
Gastrectomy	2	2
Myomectomy	2	4
Ovarian cyst	8	4
Retroperitoneal mass total abdominal hysterectoomy	2	0
Hysterectomy	17	25

Although on the first day the grade 2 of nausea was not observed in any of the patients in both groups, some in the control group suffered from grade 2 of nausea at T_0_ + 24, 32, 40 and 48 hours. The difference was significant after 24 and 48 fours (P values = 0.016 and 0.038) but it was not significant at T_0_+32 and 40 (P values = 0.213 and 0.082). In each of the two groups 5 patients had taken one dose of ondansetron on the first day.

Our evaluation at 8-hour intervals (up to 48 hours) showed no significant difference in the incidence of itching among the patients of both groups and no neurological and psychological complications were observed. Assessment of the sedation score at T_0_, T_0_ + 1, 8, 16, 24, 32, 40 and 48 hours indicated no significant difference between the two groups. However, at T_0_ + 16 hours, 5 patients in the ketamine group had become agitated (Ramsay score 1). At this time the other patients in ketamine group and all subjects in the control group were located in Ramsay score 2. Therefore sedation score at this time turned out to be significant in Chi-square test (P = 0.022) but not significant in Fisher’s exact test (P = 0.056). 

## 5. Discussion

In this study the effects of adding ketamine to intravenous fentanyl plus acetaminophen PCA were investigated, which revealed no significant differences in pain scores between the control and ketamine group (while resting), moving and coughing, at T_0_,T_0_ + 8, 16, 24, 32, 40 and 48 hours.

When NMDA antagonist administration is accompanied with an opioid in animals, a synergistic or additive analgesic effect is observed. This could lead to administration of reduced doses of both drugs and lesser consequent side effects. Studies carried out on animals indicated that NMDA antagonists block the course of resistance to continuous morphine exposure; in fact opioid-induced resistance is decreased and reversed (11).

Taking opioids usually resulted in side effects such as nausea, heavy sedation and rarely respiratory depression. Resistance to the analgesic effect can rise even during the early stages of treatment with opioids (11).

In one study, the addition of ketamine to tramadol after major abdominal surgeries resulted in more relief in pain and less tramadol consumption (16).

A combination of analgesia and synergisms is probably the reason for this effect and it is reinforced by taking advantage of lower PCA bolus doses in the ketamine and magnesium groups that have the same VRS scores. It is also likely to reach a synergistic effect of tramadol with magnesium or ketamine (16).

In a survey, administration of ketamine has relieved the postoperative pain after laparotomy (17). Intramuscular administration of ketamine (0.44 mg/kg) can induce analgesia for experimental pain or pain after surgery but it will have a quick start and will last for a fairly short period of time. The main observed side effect of the mentioned ketamine dose was slight dizziness and the psychic effects that were not only concerning but also appeared to improve the drug clinical efficiency (17).

Unlugenc and colleagues found that the addition of ketamine to morphine in intravenous PCA relieved the pain and reduced morphine consumption (18). In a study on pain control after tonsillectomy, the sprays of lidocaine, morphine and ketamine were applied at the end of surgery. After 20 minutes, the lidocaine spray was more effective than other two drugs while after 40 minutes the sprays of ketamine and morphine were more effective (19).

Several studies are available in the field of managing abdominal postoperative pain which investigated the effects of adding ketamine to opioids (20).

Evaluating the negative results of the study, some influences must also be taken into account; these factors include administration method, the number of patients, stability of ketamine-morphine solution, concentration ratio of ketamine-morphine solution, the design of the study, ketamine dose, pain type and pharmacokinetics (20).

Eight placebo-controlled studies investigated the effects of intravenous and intramuscular ketamine on postoperative pain and 7 of them showed analgesic effects (21).

Different ketamine doses (4 - 30 mg) have been experimented epidurally but a small number of these studies have been carried out in randomized, double-blind trial and placebo-control forms (21).

Sveticic examined twelve different combinations of morphine and ketamine. The least severe pain and the least adverse effects were achieved with a ketamine/morphine ratio of 1:1 and at a bolus dose of 0.7 - 1.8 mg of each drug repeated every 8 minutes (22).

It is possible that various bolus dose amounts stem from the familiar interindividual differences in the required drug amount in order to benefit from adequate analgesia. Lack of consistency in lockout times does not seem to impose an effect on the pain scores generally (22).

Studies carried out by Reeves do not reinforce the theory that lower doses of ketamine can have a beneficial role in boosting morphine PCA in patients while they are in post abdominal surgery recovery. Nevertheless, lower doses of ketamine are proved to possess a number of influences that can be recognized clinically, especially reduced cognitive performance. Long period sleep was another result but statistically it is not significant. Therefore the ketamine dose examined in the present study was sufficient. Higher doses of ketamine might lead to notable results but most probably will bring about more problems (23).

In those studies the ketamine has increased the analgesia, the initial low-dose (0.125 - 2 mg/kg) had usually been used but it has not been effective in those studies where the initial dose had not been administered (18).

The present study tried to minimize the side effects of the drugs by reducing the total dose of fentanyl and acetaminophen as a continuous infusion in PCA. To compensate for the reduced total dose of fentanyl and acetaminophen, ketamine was administered as a concomitant drug to increase the analgesic effects (24).

Generally, the pain score at the beginning was larger in the ketamine group compared with the control group. 

In this study we did not measure the ketamine plasma levels. When fentanyl is combined with ketamine the electrical current pain threshold dose response curve is substantially sharper than only fentanyl dose response curve. 30 ng/mL concentration of ketamine serum in itself made no modifications in electrical pain threshold but when added to fentanyl, it led to a larger rise in pain threshold compared to that of fentanyl in itself. Ketamine did not enhance the anti-nociceptive features of fentanyl in the assessment of nociception stimulation (with heat and pressure). Subjective and objective measurements of sedation indicated no differences between fentanyl sedative effect and that of fentanyl combined with ketamine. The administered doses of drug in the study at hand did not affect cardiovascular and respiratory factors. 

Anti-nociceptive features of fentanyl were enhanced with a serum concentration of ketamine that had made no changes on sedation levels. The improvement of anti-nociceptive features was followed by no rise in sedation. This is a sign that in order to increase the analgesic effect in a clinic, lesser continuous doses of ketamine (30 - 120 ng/mL) can be combined with μ opioid agonists (25).

At T_0_, T_0_ + 8, 16, 24, 32, 40 and 48 hours, no significant differences in pain scores were observed (while resting, moving and coughing) between the control and ketamine group. 
